# Single-Cell RNA-seq Analysis Reveals a Positive Correlation between Ferroptosis and Beta-Cell Dedifferentiation in Type 2 Diabetes

**DOI:** 10.3390/biomedicines12081687

**Published:** 2024-07-29

**Authors:** Jiajing Ma, Xuhui Li, Xuesi Wan, Jinmei Deng, Yanglei Cheng, Boyuan Liu, Liehua Liu, Lijuan Xu, Haipeng Xiao, Yanbing Li

**Affiliations:** 1 Department of Endocrinology and Diabetes Center, The First Affiliated Hospital of Sun Yat-sen University, 58 Zhongshan Road II, Guangzhou 510080, China; majj8@mail2.sysu.edu.cn (J.M.); lixh56@mail2.sysu.edu.cn (X.L.); wanxues@mail.sysu.edu.cn (X.W.); chengylei@mail2.sysu.edu.cn (Y.C.); liuby33@mail2.sysu.edu.cn (B.L.); liulieh@mail.sysu.edu.cn (L.L.); xulijuan@mail.sysu.edu.cn (L.X.); xiaohp@mail.sysu.edu.cn (H.X.); 2 Internal Medicine Department, The First Affiliated Hospital of Sun Yat-sen University, 58 Zhongshan Road II, Guangzhou 510080, China; dengjm26@mail2.sysu.edu.cn

**Keywords:** ferroptosis, dedifferentiation, type 2 diabetes mellitus, single-cell sequencing, machine learning, bioinformatics

## Abstract

Insulin deficiency in patients with type 2 diabetes mellitus (T2D) is associated with beta-cell dysfunction, a condition increasingly recognized to involve processes such as dedifferentiation and apoptosis. Moreover, emerging research points to a potential role for ferroptosis in the pathogenesis of T2D. In this study, we aimed to investigate the potential involvement of ferroptosis in the dedifferentiation of beta cells in T2D. We performed single-cell RNA sequencing analysis of six public datasets. Differential expression and gene set enrichment analyses were carried out to investigate the role of ferroptosis. Gene set variation and pseudo-time trajectory analyses were subsequently used to verify ferroptosis-related beta clusters. After cells were categorized according to their ferroptosis and dedifferentiation scores, we constructed transcriptional and competitive endogenous RNA networks, and validated the hub genes via machine learning and immunohistochemistry. We found that ferroptosis was enriched in T2D beta cells and that there was a positive correlation between ferroptosis and the process of dedifferentiation. Upon further analysis, we identified two beta clusters that presented pronounced features associated with ferroptosis and dedifferentiation. Several key transcription factors and 2 long noncoding RNAs (*MALAT1* and *MEG3*) were identified. Finally, we confirmed that ferroptosis occurred in the pancreas of high-fat diet-fed mice and identified 4 proteins (NFE2L2, CHMP5, PTEN, and STAT3) that may participate in the effect of ferroptosis on dedifferentiation. This study helps to elucidate the interplay between ferroptosis and beta-cell health and opens new avenues for developing therapeutic strategies to treat diabetes.

## 1. Introduction

Type 2 diabetes mellitus (T2D) is a widespread chronic metabolic disease characterized by insulin resistance and decreased insulin production, resulting in elevated blood glucose levels [1]. It represents a significant global health concern that is increasing in incidence and prevalence rates. The International Diabetes Federation estimates that T2D may affect up to 783.2 million individuals worldwide by 2045, up from 536.6 million in 2021 [2]. In addition, T2D is associated with a multitude of complications [3], including cardiovascular disease, neuropathy, nephropathy, and retinopathy, significantly impacting both individual health and public health care systems. Progressive beta-cell dysfunction is generally known to be a major factor in the development of T2D [4,5], and multiple observations, such as decreased beta-cell mass, beta-cell exhaustion, and dedifferentiation or transdifferentiation into different cell types, have been used to clarify this process [6,7]. Despite ongoing research, the precise mechanisms behind beta-cell dysfunction in T2D remain elusive. Gaining molecular insight into beta-cell disorders could enhance approaches for treating patients with T2D.

Dedifferentiation of beta cells is characterized by diminished expression of beta-cell-enriched genes and the concomitant upregulation of other genes including markers of progenitor cells or other islet endocrine cells [8]. These changes cause beta cells to reorganize metabolically and structurally, which results in insufficient insulin secretion [9]. Although past and current studies have demonstrated a significant contribution of hyperglycemia and the resulting glucotoxicity to beta-cell dedifferentiation [10], investigations are still ongoing to determine the specific molecular processes involved. Elucidating these mechanisms broadens new treatment possibilities for preserving or restoring functional beta cells in T2D.

Ferroptosis is characterized by an imbalance in a cell’s labile iron levels, resulting in the accumulation of iron-dependent lipid peroxidation products and disrupted redox equilibrium [11]. Over the years, iron-dependent cell death has been extensively investigated across various pathogenic processes from development and aging to immunity and cancer [12]. An increasing number of researchers are studying the impact of ferroptosis on beta cells [13]. According to one study, an increased risk of T2D may be attributed to excessive body iron storage [14]. A Mendelian randomization study revealed a causal relationship between elevated systemic iron status and heightened risk of T2D [15]. Recent studies have also revealed the involvement of ferroptosis in beta-cell dysfunction and death. Dysregulated iron metabolism and oxidative-stress-induced lipid peroxidation have been observed in beta cells under conditions associated with T2D, such as hyperglycemia, streptozotocin, and hydrogen peroxide [16]. Ferrostatin 1, a ferroptosis inhibitor, reduced these effects and prevented cell death. In addition, iron overload increases fasting blood glucose and impairs insulin expression in mice [17]. These results indicate that ferroptosis may participate in the dysfunction of T2D beta cells. 

Single-cell RNA sequencing (scRNA-seq), which can identify genes in numerous types of cells, has recently gained popularity. Here, via scRNA-seq data analysis, we revealed a connection between ferroptosis susceptibility and dedifferentiation in beta cells, and we also revealed the existence of ferroptosis-related beta clusters. We constructed networks for key transcription factors and differentially expressed genes (DEGs) as well as competitive endogenous RNAs (ceRNAs) via differentially expressed long noncoding RNAs (lncRNAs) to identify possible regulatory pathways involved in the pathological mechanism of T2D. We subsequently established diabetic mouse models using a high-fat diet regimen and confirmed the occurrence of ferroptosis in the pancreas of these mice. Finally, we identified eight hub genes in beta cells via Least Absolute Shrinkage and Selection Operator (LASSO) and Random Forest (RF) algorithms and verified the expression of 4 of them in the T2D mouse model. Overall, this research contributes to our understanding of the molecular basis and influence of ferroptosis in beta cells.

## 2. Materials and Methods

### 2.1. Data Acquisition

The scRNA-seq data from individuals with T2D used in this study were obtained from the online public databases GEO and ArrayExpress, including 5 GEO datasets (GSE124742 [18], GSE154126 [19], GSE81608 [20], GSE86469 [21], GSE98887 [22]) and 1 ArrayExpress dataset (E-MTAB-5061 [23]). We converted the raw data from high-throughput sequencing into transcripts per million (TPM) values for each sample. R x64 4.2.1 and associated R Bioconductor packages were used to analyze all of the data. From the FerrDb online database (http://www.zhounan.org/ferrdb/current/, accessed on 24 July 2024) [24], we were able to obtain 445 ferroptosis-related genes (FRGs). 

### 2.2. Quality Control, Cluster Analysis, and Cell Type Annotation of scRNA-seq Data

We converted scRNA-seq data into Seurat objects and removed batch effects with the aid of the “Seurat” package (version 4.3.0.1). Two quality indicators were applied to the raw matrix for each cell: percentage of ERCC genes (percent.ERCC < 5%), and percentage of red blood cell genes (percent.HB < 0.25%). The top 2000 highly variable genes were subjected to principal component analysis (PCA) to determine the most significant principal components (PCs) after quality control and data filtering. Then, objective visualization of cell subpopulations was accomplished by applying Uniform Manifold Approximation and Projection (UMAP). The “FindAllMarkers” function was used to compare the variations in gene expression between clusters. Additionally, for cell type annotation, we employed the following markers: for beta cells, *INS*; for alpha cells, *GCG*; for delta cells, *SST*; for PP cells, *PPY*; for acinar cells, *PRSS1*; for ductal cells, *KRT19*; for stellate cells, *SPARC*; and for immune cells, *LATPM5* [23]. The co-expressing cells were identified as expressing both *INS* and *GCG*, while the cells without clear markers were identified as unknown cells. Then, we extracted all beta cells and applied a similar procedure for subcluster analysis, including variable gene identification, dimension reduction, and clustering visualization.

### 2.3. Identification of DEGs, DELs, and Ferroptosis-Related DEGs

To identify DEGs and differentially expressed long non-coding RNAs (DELs) between different groups of beta cells, the Wilcoxon rank-sum test, which was implemented using the “FindMarkers” function of the “Seurat” package, was used to perform differential expression analysis. Genes that met the cut-off criteria of a *p*-adjusted value < 0.05 and |Log2FC| > 0.5 were considered to be DEGs and DELs. To find DEGs associated with ferroptosis, we examined the intersection of FRGs and DEGs.

### 2.4. Gene Set Enrichment Analysis (GSEA) and Score Calculation

GSEA was used to explore the implication of the “WP_FERROPTOSIS” pathway in T2D and non-diabetic beta cells using the “fgsea” package (version 1.24.0) with C2 gene sets (c2.all.v2023.1.Hs.symbols.gmt) from the Molecular Signatures Database (MSigDB, v2023.1.Hs, http://software.broadinstitute.org/gsea/msigdb, accessed on 24 July 2024) [25]. To calculate scores for ferroptosis- and dedifferentiation-related gene expression in single cells, the “AddModuleScore” function of the “Seurat” package was applied. We selected 17 genes as dedifferentiation markers, including 9 progenitor-cell markers (*NEUROG3*, *GATA6*, *HNF4A*, *NOTCH1*, *HES1*, *OCT4*, *NANOG*, *ALDH1A3*, *L-MYC*) and 8 alpha-cell markers (*GCG*, *ARX*, *IRX2*, *MAFB*, *POU6F2*, *FEV*, *KCNJ3*, *SV2B*) [10]. FRGs from the Ferroptosis Database were used as ferroptosis markers. Based on the median scores for ferroptosis and dedifferentiation, we separated all beta cells into four categories: Ferro_high_Dediff_high (double-high), Ferro_high_Dediff_low, Ferro_low_Dediff_high, and Ferro_low_ Dediff_low (double-low).

### 2.5. Gene Set Variation Analysis (GSVA)

GSVA is an unsupervised, non-parametric technique that uses sample gene expression matrix data to estimate changes in gene set enrichments [26]. Using the “GSVA” package in R (version 1.48.3), an enrichment score was generated for each sample and pathway to transform the gene expression data into a matrix of gene set expression. To calculate each pathway gene set, the Kolmogorov–Smirnov test statistic was employed.

### 2.6. Pseudo-Time Trajectory Analysis

Pseudo-time trajectory analysis was performed using Monocle 3 (version 1.3.1) [27]. The 6 beta-cell clusters identified in subcluster analysis were used as 6 trajectories. The root cluster was designated as Cluster 0. Gene expression was first normalized via the “estimate_size_factors” function, and then variations in gene expression over pseudo-time were examined using the “graph_test” function. 

### 2.7. Transcription Factor Activity Inference and Network Construction

Transcription factor (TF) activity was inferred using the DoRothEA resource (dorothea_1.7.3, https://saezlab.github.io/dorothea/, accessed on 24 July 2024) [28]. We built regulons using the mRNA expression levels of each TF’s direct targets as well as the expression levels of each TF from a manually selected database. Using the “dorothea_regulon_human” wrapper function from the “DoRothEA” library (version 1.12.0), we generated TF regulons and selected “A”, “B”, and “C” as high-confidence TF choices. VIPER scores were added to the integrated Seurat object as the “DoRothEA” slot after being calculated on DoRothEA regulons and scaled. The scaled VIPER scores’ mean and standard deviation were calculated for each group to enable comparison of the TF score activities. The variance of the corresponding VIPER scores was used to rank the TFs. For the purpose of visualizing the corresponding scores, the top 15 highly variable scores for each group (a total of 30 TFs) were retained. The Transcriptional Regulatory Relationships Unraveled by Sentence-based Text mining (TRRUST) database (TRRUST v2, https://www.grnpedia.org/trrust/, accessed on 24 July 2024) was utilized in our study to analyze TFs linked to target genes [29]. Subsequently, we created a TF and target gene network in Cytoscape (version 3.9.1).

### 2.8. Construction of a ceRNA Network

TargetScan Human (version 7.2) (http://www.targetscan.org/vert_72/, accessed on 24 July 2024), miRDB (version 6.0) (http://mirdb.org/, accessed on 24 July 2024), miRWalk 3.0 (http://mirwalk.umm.uni-heidelberg.de/, accessed on 24 July 2024), and starBase (version 2.0) (https://rnasysu.com/encori/, accessed on 24 July 2024) with default parameters were employed to forecast mRNA–miRNA interactions. StarBase (version 2.0) was used to predict lncRNA–miRNA interactions. The ceRNA network was constructed and visualized utilizing the “ggalluvial” package in R (version 0.12.5).

### 2.9. Screening Key Genes Associated with T2D Using Machine Learning Techniques

The machine learning algorithm known as Least Absolute Shrinkage and Selection Operator (LASSO), which combines the features of ridge regression and subset selection, is frequently used to identify the best variables by determining the lambda value at which the classification model error is the lowest [30]. The LASSO regression analysis was carried out using the “glmnet” package (version 4.1-7) [31]. In order to screen for important genes, the expression of candidate genes was examined using LASSO regression with a binomial model and a lambda value equal to the minimum mean cross-validated error. 

To find and validate possible predictors, another machine learning algorithm called Random Forest (RF) was used. RF is based on building many decision trees and is capable of training and predicting samples with high accuracy [32]. Here, we confirmed hub genes independently using the “randomForest” package (version 4.7-1.1). The mean decrease accuracy and mean decrease Gini (MDG) were calculated to evaluate the classification performance of key genes.

### 2.10. Correlation Analysis, Expression Level, and AUC Calculation of Key Genes

Pearson correlation analysis was implemented to determine the relationships between the key genes. The correlation coefficient, or “r value,” was used in this type of correlation analysis to assess the effect measurement. Then, a correlation matrix heatmap was generated using the R function “corrplot”. Using the Wilcox test, the expression levels of key genes were contrasted between T2D and non-diabetic samples; a *p* value < 0.05 was deemed statistically significant. The receiver operator characteristic (ROC) curves were drawn using the “pROC” package (version 1.18.2), and areas under the curve (AUCs) were computed to assess the significance of key genes for diagnosis.

### 2.11. T2D Mouse Model

Male C57BL/6J DIO 60% (Diet-Induced Obesity) mice and control mice of the same sex and age (*n*  =  13/group) were purchased from GemPharmatech (Nanjing, China). To create a T2D model, 5-week-old male C57BL/6J mice were fed a high-fat diet (HFD) regimen (60% of energy from fat, Research Diets, Inc., New Brunswick, NJ, USA), whereas their control counterparts were fed a chow diet (18% of energy from fat). After 12 weeks of a 60% high-fat diet or chow diet, the mice were euthanized, and their pancreases were extracted for subsequent experiments. The study protocol was approved by the Animal Care and Use Committee of Sun Yat-sen University (Approval Number: SYSU-IACUC-2024-000265).

### 2.12. Determination of Biochemical Parameters

After fasting the mice for 6 h, we measured fasting blood glucose using the tail-clip method. An intraperitoneal (i.p.) glucose tolerance test (GTT) was performed on 16 h overnight-fasted mice. Blood glucose was assessed at the indicated times using an Accu-Chek Active glucometer (Roche Diagnostics GmbH, Basel, Switzerland). Iron levels in both serum and pancreatic homogenates were determined using the Iron Assay Kit (Biosharp BL898B, Hefei, China) following the manufacturer’s protocol. Serum ferritin levels were quantified with the Mouse Ferritin ELISA Kit (Elabscience E-EL-M0491c, Wuhan, China). Additionally, we measured pancreatic homogenate levels of malondialdehyde (MDA) and glutathione (GSH) using the Lipid Peroxidation MDA Assay Kit (Biosharp BL904A, Hefei, China) and the GSH Assay Kit (Solarbio BC1170, Beijing, China), respectively.

### 2.13. Immunohistochemistry (IHC)

An IHC experiment was performed on the pancreas to detect the expression of key genes. Pancreas specimens were immersed in paraffin after being cleaned in PBS and fixed with 4% paraformaldehyde. After a one-hour incubation period at 65 °C, paraffin sections were deparaffinized using xylene and hydrated in ethanol at decreasing graded concentrations. Sections were boiled in sodium citrate buffer (10 mM, pH 6.0) for three minutes in a pressure cooker to retrieve the antigen. An inhibitor of peroxidase was used to inhibit endogenous peroxidase activity (Servicebio, Wuhan, China). Following blocking with PBS buffer containing 5% bovine serum albumin, the sections were incubated with specific antibodies (LAMP2 antibody: Servicebio GB11330; NFE2L2 antibody: Servicebio GB113808; STAT3 antibody: affinity #BF0354; SCP2 antibody: ABCAM ab140126; CHMP5 antibody: affinity #DF14834; ARF6 antibody: affinity #DF6170; CHP1 antibody: SAB #47772; PTEN antibody: Servicebio GB113803) at 4 °C overnight before a 20-min room temperature incubation with a secondary antibody (Servicebio, Wuhan, China). Hematoxylin (Servicebio, Wuhan, China) was used as a counterstain after sections were stained with 3,3′-diaminobenzidine (Servicebio, Wuhan, China). The analysis of the images was carried out by ImageJ software (version 1.52a).

### 2.14. Statistical Analysis

GraphPad Prism software (verison 9.5) was used to analyze the data of mouse models, and the results were displayed as means ± standard deviations. The unpaired Student’s *t*-test was used to analyze the group differences. It was determined that a *p* value of less than 0.05 was statistically significant.

## 3. Results

### 3.1. Data Collection, Quality Control, and Cell Clustering

**Figure 1:**
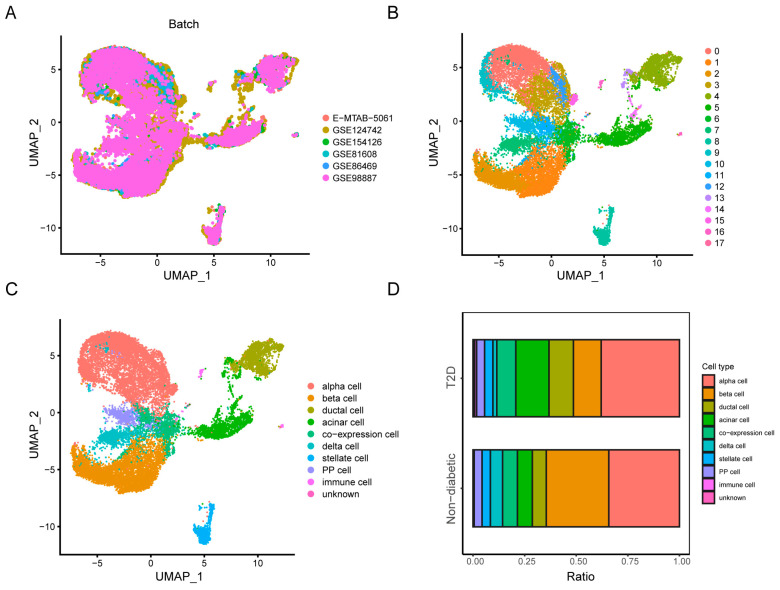
Integration, clustering, and cell proportion calculation of single-cell RNA sequencing (scRNA-seq) data. (**A**) Uniform Manifold Approximation and Projection (UMAP) plot showing elimination of batch effect. (**B**) Eighteen clusters visualized based on UMAP. (**C**) Cell populations identified by marker genes. (**D**) Comparison of cell proportions between type 2 diabetes mellitus (T2D)-affected islets and non-diabetic islets.

ScRNA-seq analysis was performed on 85 samples from six scRNA-seq datasets. The dataset information is shown in Supplementary Table S1. After initial quality control tests were completed for each dataset, we performed data integration with the “Seurat” package to eliminate batch variance (Figure 1A). The violin plots display the overall number of genes (nFeature_RNA), RNA expression amounts for genes (nCount_RNA), percentage of mitochondrial genes (percent.MT), percentage of ERCC genes (percent.ERCC), percentage of ribosomal genes (percent.RB), and percentage of erythrocyte-related genes (percent.HB) in each dataset (Supplementary Figure S1A). According to the plots, we used percent.ERCC < 5 and percent.HB < 0.25 as cutoffs to filter cells. Supplementary Figure S1B shows the gene numbers detected, and the expression values of the genes were proportional to each other. We then used PCA to reduce the dimensionality and visualize the distribution as a whole. In this work, we selected 20 principal components and set the resolution at 0.5 for cell clustering (Supplementary Figure S1C,D). As the UMAP plot shows, 18 different clusters were found among 19644 cells (Figure 1B). After cell annotation, different types of endocrine and nonendocrine cells were clearly classified and were characterized using specific markers (Supplementary Figure S2A). The distribution of each cluster and the locations of the corresponding marker genes were consistent, suggesting the validity of the cluster analysis and the identification of the primary cell types. The dot map illustrates the relative expression of three cluster marker genes in each cell type (Supplementary Figure S2B). Consistent with previous findings, the number of beta cells in T2D islets decreased compared with control samples from 30.3% to 13.5%, and the number of alpha cells in T2D islets increased from 34.2% to 37.9% (Figure 1D).

### 3.2. The Landscape of Ferroptosis in T2D

**Figure 2:**
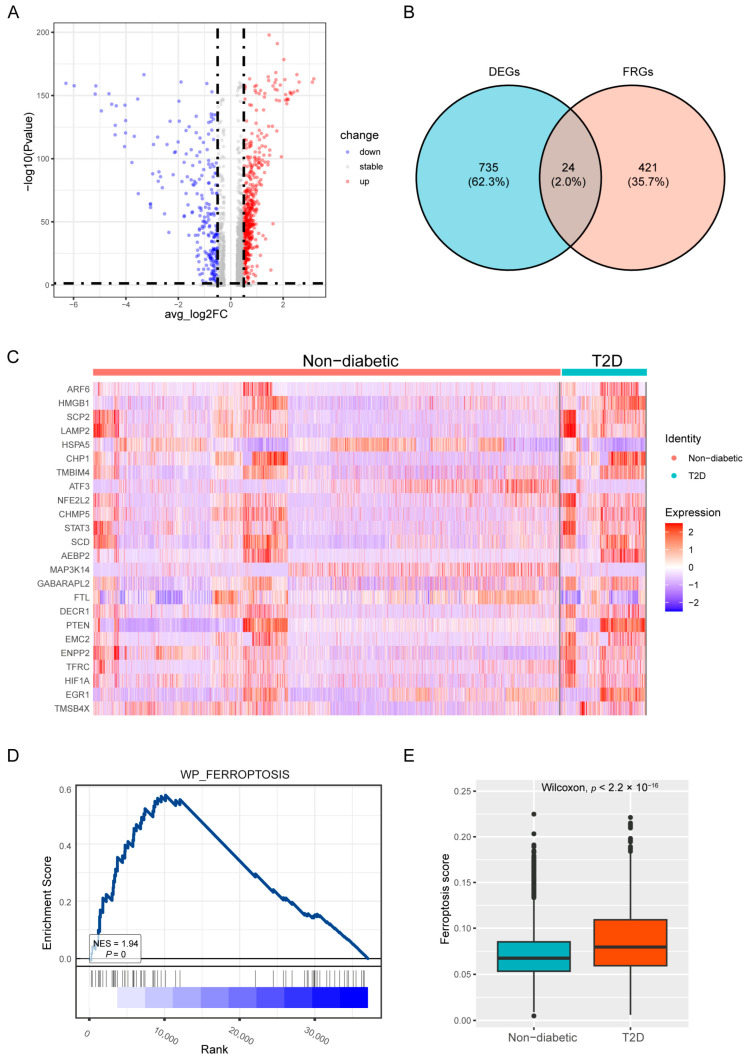
The landscape of ferroptosis in T2D. (**A**) Volcano plot of differentially expressed genes (DEGs). (**B**) Venn diagram of DEGs and ferroptosis-related genes (FRGs) based on the FerrDb database. (**C**) Heatmap of the 24 ferroptosis-related DEGs. (**D**) Gene set enrichment analysis (GSEA) of the “WP_FERROPTOSIS” pathway. (**E**) Ferroptosis scores of T2D and non-diabetic samples.

To further analyze beta cells, we separately selected 4990 beta cells. The differentially expressed genes (DEGs) between T2D and non-diabetic beta cells were identified with a *p*-adjusted value < 0.05 and |log2FC| > 0.5 as the threshold. A total of 526 genes were upregulated, and 233 genes were downregulated (Figure 2A). Twenty-four ferroptosis-related DEGs were identified when we examined the intersection of the 445 FRGs and the DEGs; twenty of these DEGs were elevated and four were downregulated in T2D samples (Figure 2B,C). In addition, according to GSEA, “WP_FERROPTOSIS” was upregulated and enriched at a *p* value < 0.05 in the T2D islets (Figure 2D). The ferroptosis score was greater in T2D beta cells than in control samples according to the “AddmoduleScore” function (Figure 2E).

### 3.3. Dedifferentiation and Ferroptosis Were Positively Correlated in Beta Cells

**Figure 3:**
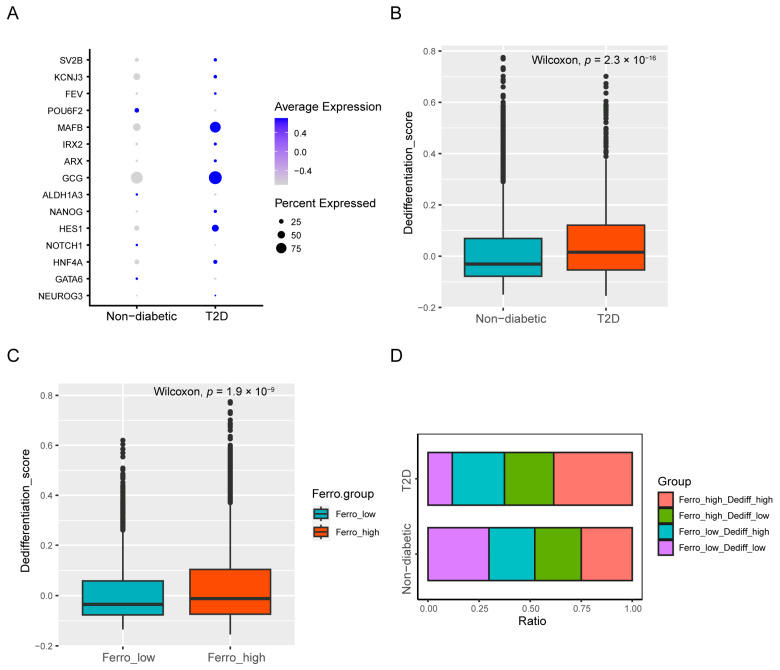
Dedifferentiation characteristics of beta cells. (**A**) Dot map of dedifferentiation-related genes. (**B**) Dedifferentiation scores of T2D and non-diabetic samples. (**C**) Dedifferentiation scores of “Ferro_high” and “Ferro_low” beta cells. (**D**) Proportion of 4 groups of beta cells between T2D and non-diabetic samples.

We assessed several progenitor-islet-cell markers and alpha-cell markers that were identified in previous research and we found that most of them were upregulated in T2D beta cells (Figure 3A). The dedifferentiation score was also greater in T2D beta cells than in control samples (Figure 3B). To explore the relationship between ferroptosis and dedifferentiation, every beta cell was assigned to one of four groups.: “Ferro_high_Dediff_high” (double-high), “Ferro_high_Dediff_low”, “Ferro_low_Dediff_high”, or “Ferro_low_Dediff_low” (double-low). The assignment was based on the medians of dedifferentiation and ferroptosis scores. The dedifferentiation score was greater in “Ferro_high” cells than in “Ferro_low” cells (Figure 3C). The bar chart clearly revealed that up to 38.5% of T2D beta cells were double-high cells, whereas 25.0% of cells were double-high in the control samples (Figure 3D), which suggests that ferroptosis and dedifferentiation in T2D beta cells were positively correlated.

### 3.4. Identification of Ferroptosis-Related Clusters in Beta Cells

**Figure 4:**
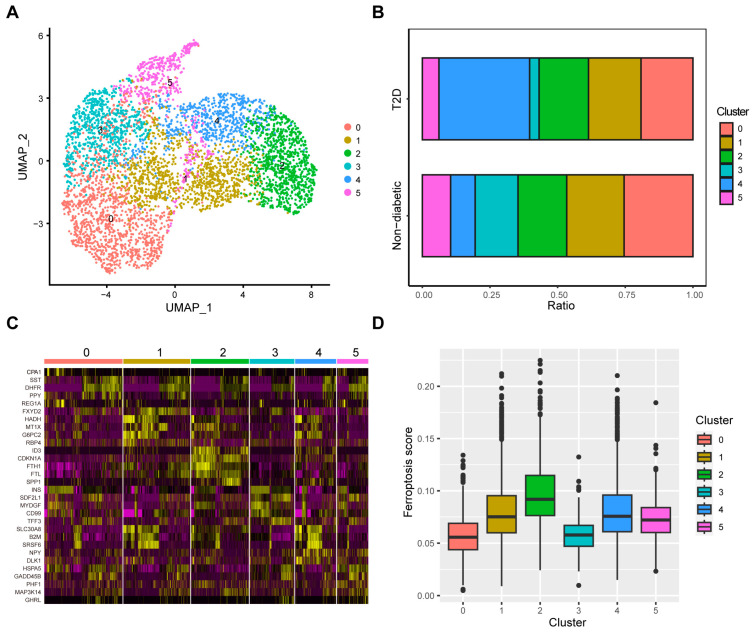
Identification of ferroptosis-related clusters in beta cells. (**A**) UMAP plot showing 6 beta-cell clusters. (**B**) Comparison of beta-cell cluster proportions between T2D and non-diabetic tissues. (**C**) Expression of 5 markers of each cluster. (**D**) Ferroptosis scores of different beta-cell clusters.

**Figure 5:**
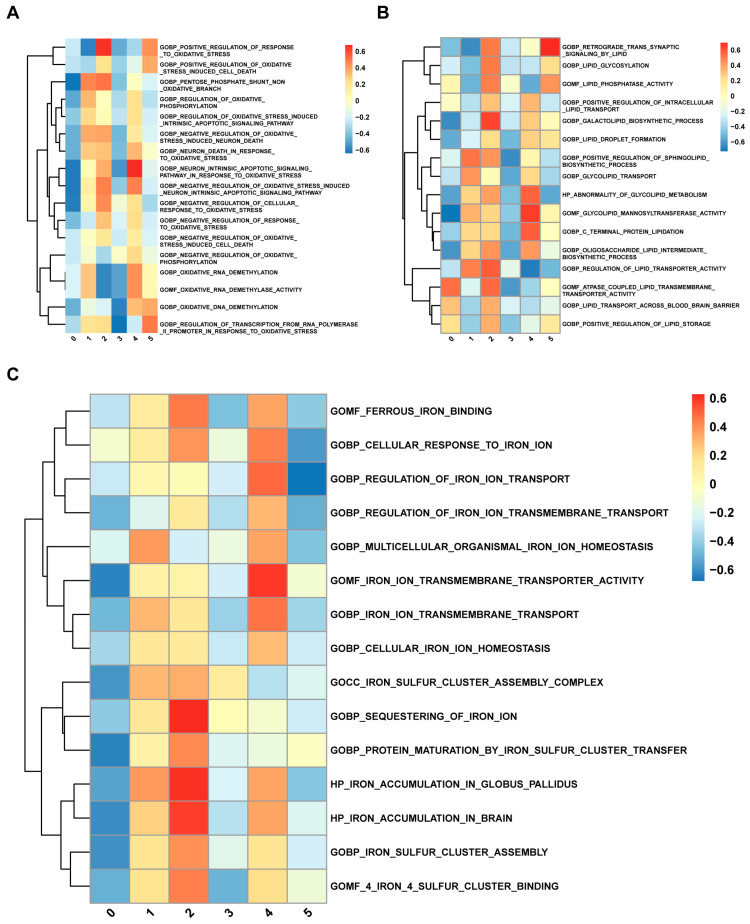
Gene set variation analysis (GSVA) of ferroptosis-related pathways in beta-cell clusters. (**A**) GSVA of oxidative-stress-related pathways. (**B**) GSVA of lipid-metabolism-related pathways. (**C**) GSVA of iron-related pathways.

Given that great heterogeneity exists among beta cells [33], we used the aforementioned methods to reduce dimensionality in beta cells via five PCs with a resolution of 0.4. We obtained six beta-cell clusters (Figure 4A). The analysis of the cell proportions suggested that Cluster 4 accounted for more diabetic islets than the other clusters (Figure 4B). Figure 4C shows the relative expression of five markers of each cluster. Ferritin genes (*FTL* and *FTH1*) were highly expressed in Cluster 2. We then calculated ferroptosis scores, and we found that Cluster 2 had the highest ferroptosis score among all the clusters and that Cluster 4 also had a relatively high ferroptosis score (Figure 4D). Furthermore, GSVA was performed to reveal pathway enrichment between clusters. The GSVA results demonstrated that pathways related to oxidative stress, lipid metabolism, and iron were enriched in Cluster 2 and Cluster 4 (Figure 5A–C).

**Figure 6:**
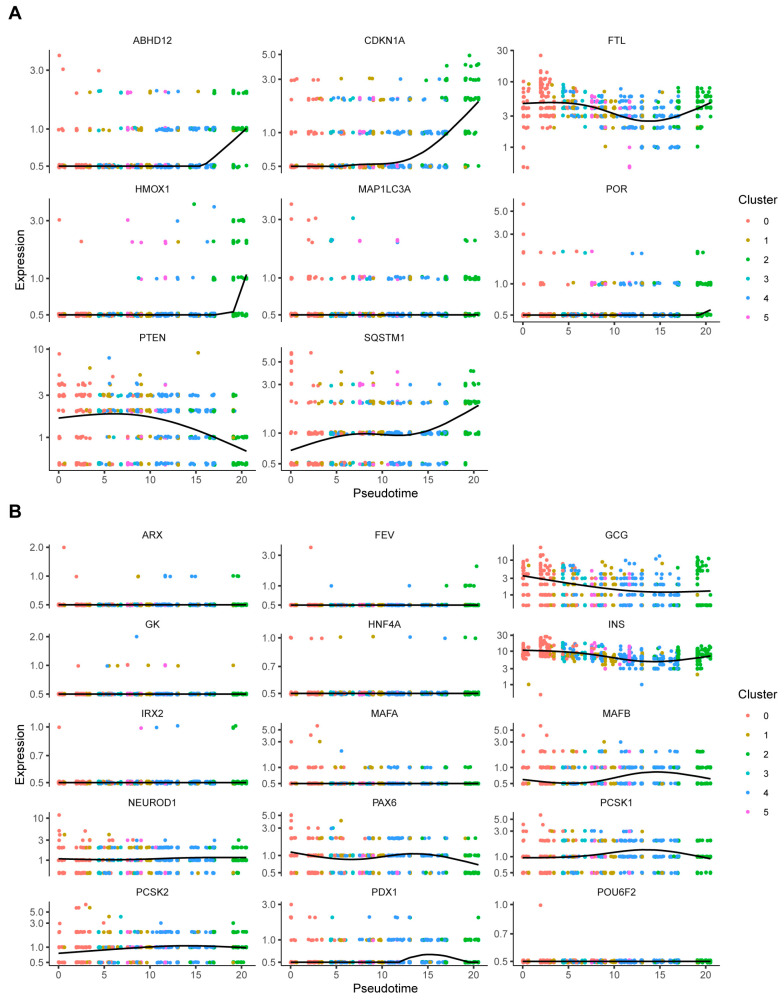
Cellular trajectory analysis in beta cells. (**A**) Pseudo-time trajectories of changes in FRGs. (**B**) Pseudo-time trajectories of changes in beta-identity-related genes.

We performed a cellular trajectory analysis to determine the differentiation states of beta-cell subpopulations and elucidate the process of change in the cells. Figure 6A,B shows the pseudo-time trajectory over 6 beta-cell clusters and Cluster 4 and Cluster 2 were located primarily at the end of the pseudo-time trajectory. In addition, the expression of FRGs and beta-identity-related genes changed from Cluster 4 to Cluster 2, indicating that the process of dedifferentiation may occur with changes in the ferroptosis process.

### 3.5. Regulatory Mechanisms of Ferroptosis in Double-High Beta Cells

**Figure 7:**
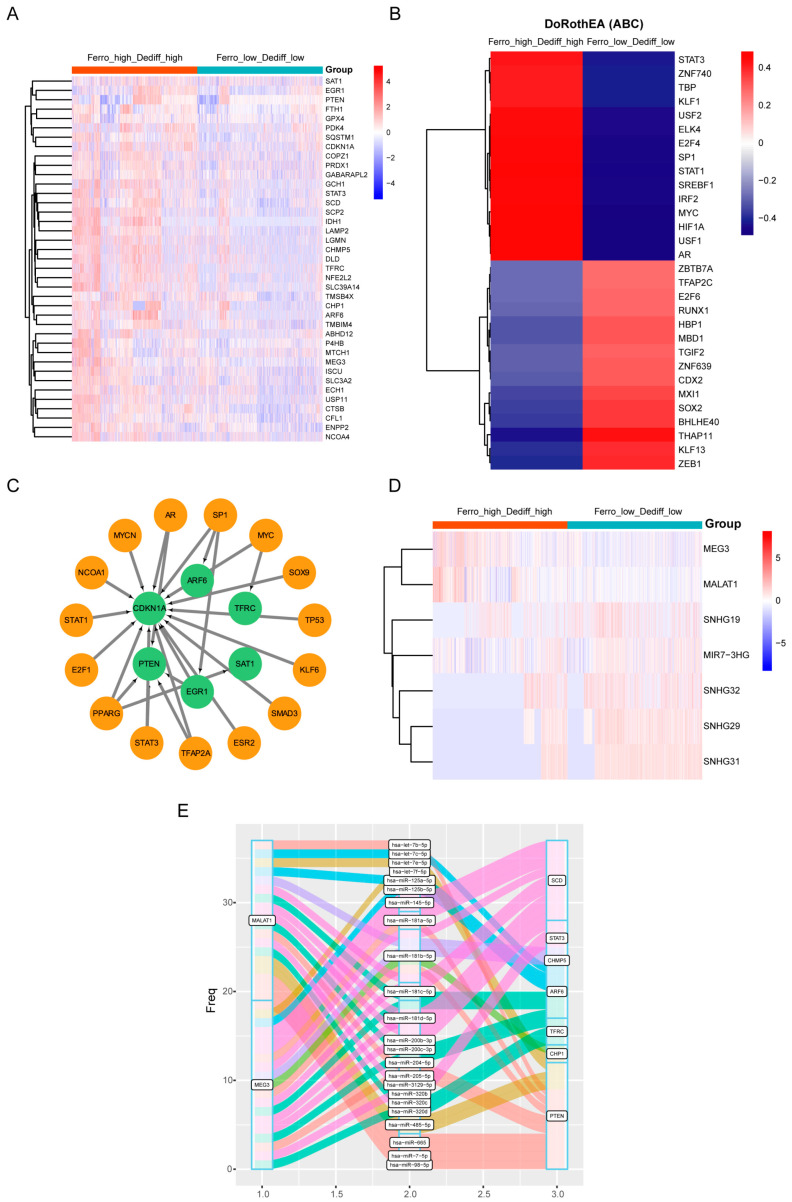
Construction of regulatory networks in beta cells. (**A**) DEGs of double-high cells compared with double-low cells. (**B**) Differentially activated transcription factors of double-high cells compared with double-low cells. (**C**) The transcriptional regulatory network (the orange circles represent TFs, and the green circles represent their target genes). (**D**) Differentially expressed long non-coding RNAs (DELs) of double-high cells compared with double-low cells. (**E**) The competitive endogenous RNA (ceRNA) network.

To further explore the mechanisms of ferroptosis in beta cells, we carried out a differential analysis of mRNAs in the double-high and double-low groups with a *p*-adjusted value < 0.05 and |log_2_FC| > 0.5 as the thresholds. A total of 39 FRGs were found to be upregulated in the double-high cells (Figure 7A). We used the DoRothEA algorithm to determine TF activity and the VIPER inference tool to score each regulon’s activity to identify upstream regulatory mechanisms. As a result, many TFs with highly variant activity levels were found. In double-high cells, we detected increased activity for TFs such as STAT3, ZNF740, TBP, KLF1, USF2, ELK4, E2F4, SP1, STAT1, SREBF1, IFR2, MYC, HIF1A, USF1, and AR (Figure 7B). The regulatory relationships between TFs and their target genes can be characterized by transcriptional regulatory networks [34]. Using the TRRUST database, we created a TF regulatory network via Cytoscape. These TFs play roles in beta cells by regulating the transcriptional activity of *CDKN1A, PTEN, EGR1, SAT1, TFRC*, and *ARF6* (Figure 7C). 

Recently, a theory has been proposed that all RNA transcripts with miRNA-binding sites can interact and regulate one another by competing for shared miRNAs, competing endogenous RNAs, or ceRNAs [35]. We identified DELs and constructed a ceRNA network. The results revealed that *MALAT1* and *MEG3* were upregulated in T2D beta cells and could regulate the expression of five FRGs (*SCD*, *STAT3*, *ARF6*, *TFRC*, *CHP1*, *PTEN*) through competitive binding to certain miRNAs (Figure 7D,E).

### 3.6. Key Genes Recognized by Machine Learning Models and ROC Analysis

**Figure 8:**
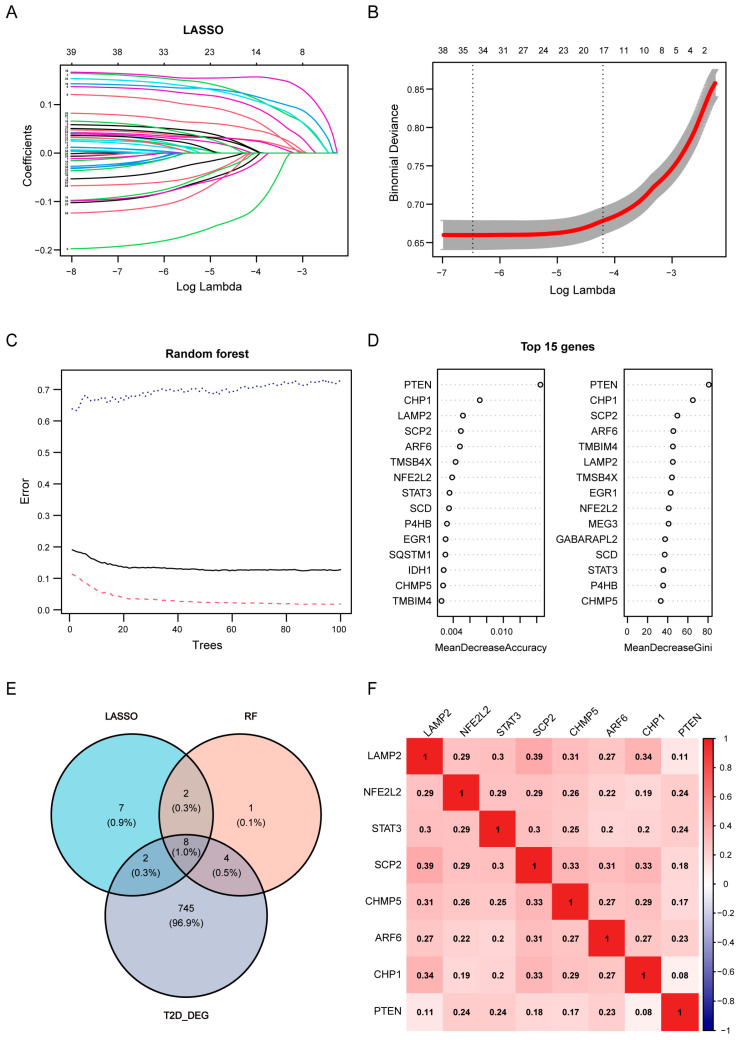
Screening of key genes using machine learning methods. (**A**) Dispersion of Least Absolute Shrinkage and Selection Operator (LASSO) coefficients. Each curve represents the trajectory of each independent variable coefficient. (**B**) Tenfold cross-validation for LASSO model parameter selection tuning. (**C**) Random Forest (RF) algorithm plot illustrating the connection between error rate and tree count. The three lines in the figure represent, from bottom to top, the error rate of the first class, the overall error rate, and the error rate of the second class, respectively. (**D**) RF algorithm-based gene ranking according to relative importance. (**E**) Venn diagram demonstrating the key genes shared by the LASSO results, RF algorithm results, and DEGs of T2D. (**F**) Correlation analysis performed among key genes.

**Figure 9:**
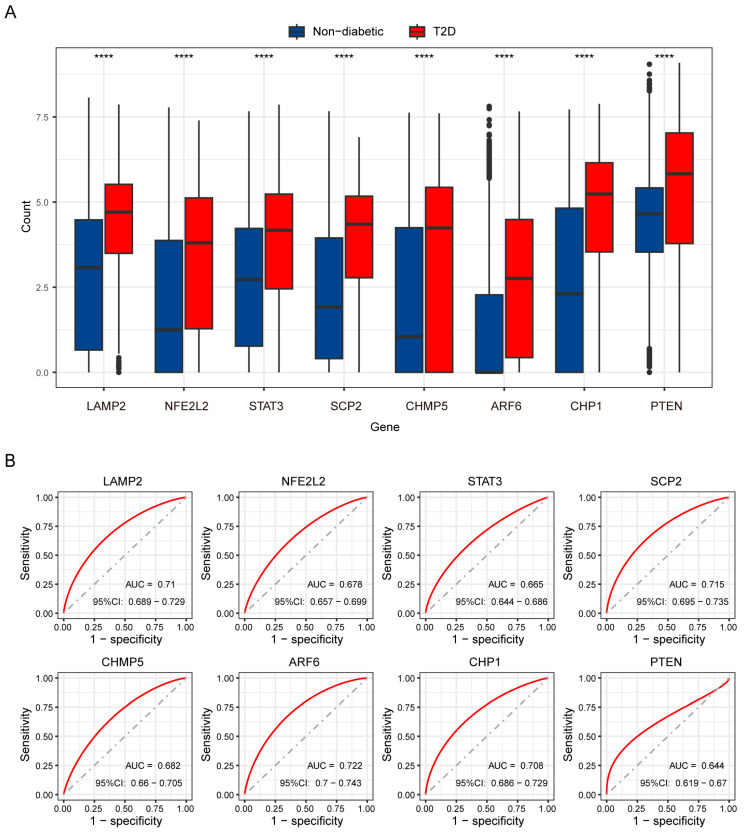
Expression and receiver operator characteristic (ROC) curves of key genes in beta cells. (**A**) Box plots displaying the expression of 8 key genes in non-diabetic and T2D samples. (**B**) ROC curves of the 8 key genes in T2D. (**** *p* < 0.0001).

To identify the highest-priority key genes, LASSO regression and the Random Forest algorithm were independently performed used to assess the 39 candidate DEGs between double-high and double-low cells. A total of 19 genes and 15 genes were identified via LASSO regression and the random forest algorithm, respectively (Figure 8A–D). Ultimately, eight genes (*LAMP2, NFE2L2, STAT3, SCP2, CHMP5, ARF6, CHP1*, and *PTEN*) overlapped among the two sets of machine learning results and the T2D DEGs, and so they were treated as key genes (Figure 8E). These key genes had relatively positive correlations with each other (Figure 8F). All of these key genes were upregulated in T2D beta cells (Figure 9A). To assess the diagnostic value of these genes, ROC curves were created and AUC values were computed. The AUC values of *LAMP2, NFE2L2, STAT3, SCP2, CHMP5, ARF6, CHP1,* and *PTEN* were 0.71, 0.678, 0.665, 0.715, 0.682, 0.722, 0.708, and 0.644, respectively, which indicates that these genes have certain diagnostic value (Figure 9B).

### 3.7. Detection of Iron Metabolism in Type 2 Diabetic Mice

**Figure 10:**
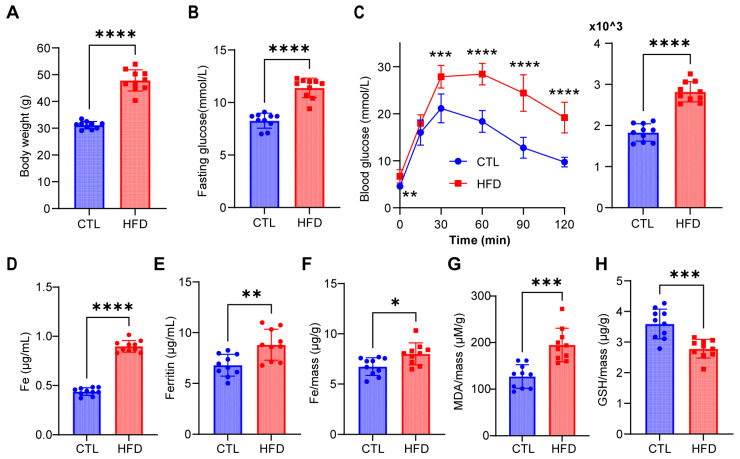
Ferroptosis markers in control and T2D mouse samples. (**A**) Body weight of control and high-fat diet(HFD) groups. (**B**) Fasting blood glucose was measured after a 6 h fast. (**C**) Glucose tolerance test (GTT) and AUC results of the control and HFD group (i.p. 2.0 g/kg glucose). (**D**) The serum iron level was measured using an iron assay kit. (**E**) The serum ferritin level was measured using a ferritin ELISA kit. (**F**) The iron level in pancreatic tissues was measured using an iron assay kit. (**G**) The malondialdehyde (MDA) level in pancreatic tissues was measured using an MDA assay kit. (**H**) The glutathione (GSH) level in pancreatic tissues was measured using a GSH assay kit. (n = 10 in each group; values are shown as means ± standard deviations. * *p* < 0.05, ** *p* < 0.01, *** *p* < 0.001, and **** *p* < 0.0001).

After 12 weeks on their respective diets, the average weight of mice in the control group was 31.16 g, whereas the average weight of mice in the HFD group was 47.88 g (Figure 10A). Individuals in the HFD group presented a fasting blood glucose level of 11.39 ± 0.9327 mmol/L, which was 37.7% higher than that of the control group (8.27 ± 0.7134 mmol/L) (Figure 10B). As shown in Figure 10C, the mice fed an HFD presented impaired glucose tolerance. These results suggest the establishment of a diabetic model through the HFD regimen.

To delve deeper into iron metabolism in type 2 diabetic mice, we measured iron levels in both serum and pancreatic tissues. Figure 10D shows a 1.05-fold increase in serum iron levels in HFD-fed mice relative to those in control mice. However, since serum iron alone is insufficient to gauge iron storage, we also assessed serum ferritin levels and found a 29.6% increase in HFD-fed mice (Figure 10E). Additionally, pancreatic iron levels were 18.7% higher in HFD-fed mice than in control mice, as depicted in Figure 10F. Collectively, these findings suggest iron overload in the diabetic pancreas.

In addition, we observed alterations in markers associated with ferroptosis in pancreatic tissues, including the antioxidant GSH and the lipid peroxidation product MDA. MDA levels were significantly elevated by 53.2% in diabetic mice (Figure 10G). Conversely, the GSH level decreased, with a 23.6% reduction relative to that in the control group (Figure 10H). These findings collectively suggest the occurrence of ferroptosis in the diabetic pancreas.

### 3.8. Validation of the Hub Genes

**Figure 11:**
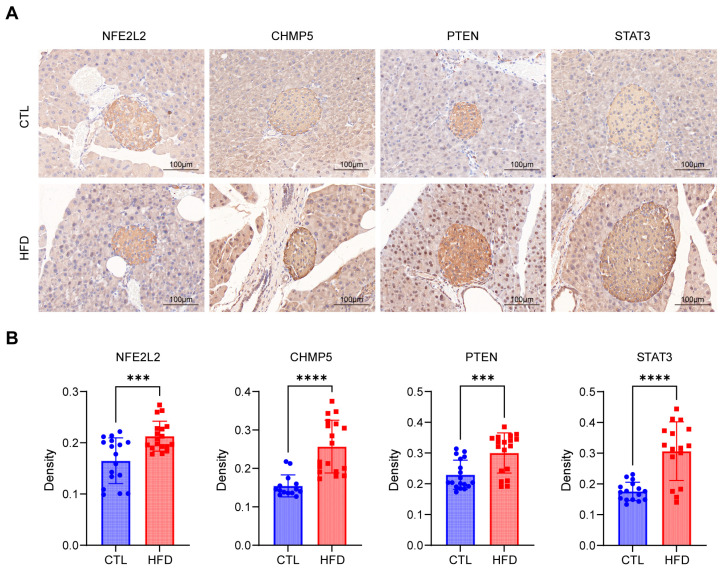
Expression of hub genes in the islets of the control and HFD groups. (**A**) Immunohistochemical staining of the NFE2L2, CHMP5, PTEN, and STAT3 proteins in the islets of the control and HFD groups. (**B**) Quantitative analysis of immunohistochemical staining of the NFE2L2, CHMP5, PTEN, and STAT3 proteins. (n = 3 in each group; 4–6 islets per mouse were analyzed; values are shown as means ± standard deviations. *** *p* < 0.001, and **** *p* < 0.0001).

IHC experiments were used to validate the protein expression levels of the eight key genes in the T2D pancreatic tissues. As shown in Figure 11A,B, the protein levels of four genes (NFE2L2, CHMP5, PTEN, and STAT3) were noticeably greater in the T2D model islets than they were in the control samples. The levels of other proteins did not significantly differ between T2D and control mice and are shown in Supplementary Figure S3A,B.

## 4. Discussion

An increasing number of studies have shown that ferroptosis may play a role in the etiology and development of T2D. A few studies have used islet sequencing data and bioinformatic analysis techniques to investigate the underlying mechanisms of ferroptosis in T2D. However, the problem is that the target gene changes cannot be located in a certain cell type, which may lead to confusing results. Thus, we employed scRNA-seq data from human tissue samples to reveal the important roles of ferroptosis in T2D at the single-cell level.

In this study, we found that relative to control cells, ferroptosis-related genes were more active in T2D beta cells and that ferroptosis scores were positively correlated with dedifferentiation scores in beta cells. We separated ferroptosis-related beta-cell clusters, which also exhibited some dedifferentiation characteristics. We subsequently explored potential upstream regulators in double-high cells by identifying key transcription factors and lncRNAs. Finally, we detected ferroptosis markers in diabetic mice and verified hub genes through machine learning and IHC.

One important factor that has been identified in the development of T2D is a decline in pancreatic beta-cell function. This decline has been linked to decreased beta-cell numbers, beta-cell exhaustion, and dedifferentiation or transdifferentiation into other kinds of cells [6]. Loss of beta-cell mass occurs during the pathogenesis of T2D [36], which was also found in our results, with beta-cell mass decreasing and alpha-cell volume increasing. In addition to cell death, dedifferentiation, which is caused by a variety of processes, including oxidative stress, endoplasmic reticulum stress, inflammation, and ROS accumulation, may contribute to the loss of functional beta-cell mass in diabetes [8]. The hallmark of ferroptosis is the iron-dependent intracellular build-up of lipid reactive oxygen species (ROS), which ultimately leads to lipid peroxidation and consequent cell death and disturbance of cellular homeostasis [12]. As ferroptosis and dedifferentiation have several common triggers, it is reasonable to associate dedifferentiation with ferroptosis. On the basis of our results, a positive link between dedifferentiation and ferroptosis can be inferred. Ferroptosis may promote dedifferentiation, and dedifferentiation may increase susceptibility to ferroptosis in beta cells. However, observational bioinformatics analysis does not yield cause-and-effect conclusions. Therefore, further research will be essential for advancing our understanding in this area.

The transcription regulatory network revealed that CDKN1A is in a central position, which suggested that it may play a vital role in certain beta-cell clusters. As a cyclin-dependent kinase inhibitor, CDKN1A inhibits the cell cycle and is regulated by multiple factors [37,38]. Recent studies have shown that CDKN1A plays protective roles in ferroptosis by promoting the activity and expression of GPX4 [39,40]. Our findings demonstrated that CDKN1A was upregulated in diabetic islets, but its effect on ferroptosis in beta cells is not clear and needs further investigation. We also analyzed DELs and identified two lncRNAs (*MALAT1* and *MEG3*). *MALAT1* decreases the histone acetylation of the *PDX1* promoter, which causes beta cells to malfunction [41]. Additionally, *MALAT1* functions as a ceRNA of miR-145-5p to control the expression of MUC1, a ferroptosis suppressor, to protect cells from ferroptosis [42]. Downregulation of Meg3 affects insulin synthesis and secretion [43]. In addition, Meg3 mediates ferroptosis by modulating p53 and inhibiting GPX4 [44]. The effects of *MALAT1* and *MEG3* on ferroptosis in beta cells also need to be verified with additional experiments.

Through machine learning and IHC, we established four hub genes (*NFE2L2*, *CHMP5*, *PTEN*, and *STAT3*) that may play a significant role in the onset of T2D. On the one hand, these hub genes are important for many domains related to ferroptosis, with NFE2L2, CHMP5, and STAT3 generally acting as ferroptosis inhibitors and with PTEN acting as a ferroptosis driver. For example, by regulating VAMP8, which promotes autophagosome-lysosome fusion, and HERC2, which is an E3 ubiquitin ligase for NCOA4 and FBXL5, NFE2L2 preserves iron homeostasis in cells [45]. Both in vitro and in vivo, human cancer cells (PANC1 and HepG2) become more susceptible to ferroptosis mediated by lipid peroxidation when CHMP5 is knocked down via RNA interference [46,47]. Loss of PTEN function confers ferroptosis resistance in cancer cells [48], and PTEN inhibitors increase hepatic NADPH, block ferroptosis, and protect the liver against ischemia/reperfusion injury [49]. *STAT3* binds to the *GPX4* promoter region and promotes *GPX4* transcription to protect cells from ferroptosis [50].

On the other hand, previous studies have identified relationships between T2D and NFE2L2, PTEN, and STAT3. In some studies, after being harmed by oxidative stress triggered by a high-fat diet, beta cells can repair themselves through the NFE2L2-related antioxidant pathway [51]. STAT3 regulates beta-cell cycling in the damaged mouse pancreas [52], guards against DNA damage, and dictates beta-cell apoptosis by modulating PTEN in streptozotocin-induced hyperglycemia [53]. Therefore, the upregulation of NFE2L2, CHMP5, and STAT3 in T2D islets may be a compensatory response to different types of stress. However, PTEN blocks cell cycle re-entry by acting on different targets, including the Akt signaling pathway and cell cycle inhibitor P16ink4a [54,55]; therefore, the upregulation of PTEN may play a pathogenic role in ferroptosis and dedifferentiation. In summary, less is known about the functions of these hub genes in the modulation of beta-cell ferroptosis, and more studies are needed to confirm their roles in T2D.

Although this study contributes to our understanding of the mechanisms underlying T2D, it has limitations. First, the data we selected were only obtained from open sources which are limited and lack the clinical information required for comprehensive analysis. Second, in cell clustering, completely dedifferentiated cells may not be identified as beta cells owing to a deficiency of specific markers, which should be explored via lineage tracing experiments. Third, we only performed IHC to confirm hub gene expression, while the biological functions of these genes were not explored via loss-of-function and gain-of-function assays; such work is the main purpose of our next study. Furthermore, the detection of only a few ferroptosis markers in diabetic models suggests that our current understanding may be incomplete. Finally, we employed only a single diabetic model to validate ferroptosis and key genes, which may differ from other animal models or human conditions.

## 5. Conclusions

In this study, we used a range of technologies to explore the relationship between ferroptosis tendency and dedifferentiation, and we explored four hub genes (*NFE2L2*, *CHMP5*, *PTEN*, and *STAT3*) as well as important transcription factors and lncRNAs that may serve as markers or treatment targets for T2D. Our research offers a new theoretical understanding of the interplay between ferroptosis and beta-cell health in T2D. Further research is needed to elucidate the intricate molecular mechanisms underlying ferroptosis and beta-cell dedifferentiation, potentially leading to novel approaches for preventing and treating T2D.

## Data Availability

The study’s original contributions are included in the article. Please do not hesitate to contact the corresponding author with any additional questions.

## Supplementary Materials

The following supporting information can be downloaded at: https://www.mdpi.com/article/10.3390/biomedicines12081687/s1, Supplementary Table S1: Data sources; Supplementary Figure S1: Basic analysis procedure for the scRNA-seq data; Supplementary Figure S2: Expression of markers in different types of endocrine and non-endocrine cells; Supplementary Figure S3: Expression of genes with no significant changes between control and T2D mouse samples.

## References

[1] Galicia-Garcia U., Benito-Vicente A., Jebari S., Larrea-Sebal A., Siddiqi H., Uribe K.B., Ostolaza H., Martin C. (2020). Pathophysiology of Type 2 Diabetes Mellitus. Int. J. Mol. Sci..

[2] Sun H., Saeedi P., Karuranga S., Pinkepank M., Ogurtsova K., Duncan B.B., Stein C., Basit A., Chan J.C.N., Mbanya J.C. (2022). IDF Diabetes Atlas: Global, regional and country-level diabetes prevalence estimates for 2021 and projections for 2045. Diabetes Res. Clin. Pract..

[3] Tomic D., Shaw J.E., Magliano D.J. (2022). The burden and risks of emerging complications of diabetes mellitus. Nat. Rev. Endocrinol..

[4] Weir G.C., Gaglia J., Bonner-Weir S. (2020). Inadequate β-cell mass is essential for the pathogenesis of type 2 diabetes. Lancet Diabetes Endocrinol..

[5] Hudish L.I., Reusch J.E., Sussel L. (2019). β Cell dysfunction during progression of metabolic syndrome to type 2 diabetes. J. Clin. Investig..

[6] Wysham C., Shubrook J. (2020). Beta-cell failure in type 2 diabetes: Mechanisms, markers, and clinical implications. Postgrad. Med..

[7] Cinti F., Bouchi R., Kim-Muller J.Y., Ohmura Y., Sandoval P.R., Masini M., Marselli L., Suleiman M., Ratner L.E., Marchetti P. (2016). Evidence of β-Cell Dedifferentiation in Human Type 2 Diabetes. J. Clin. Endocrinol. Metab..

[8] Talchai C., Xuan S., Lin H.V., Sussel L., Accili D. (2012). Pancreatic β cell dedifferentiation as a mechanism of diabetic β cell failure. Cell.

[9] Remedi M.S., Emfinger C. (2016). Pancreatic β-cell identity in diabetes. Diabetes Obes. Metab..

[10] Bensellam M., Jonas J.C., Laybutt D.R. (2018). Mechanisms of β-cell dedifferentiation in diabetes: Recent findings and future research directions. J. Endocrinol..

[11] Stockwell B.R. (2022). Ferroptosis turns 10: Emerging mechanisms, physiological functions, and therapeutic applications. Cell.

[12] Jiang X., Stockwell B.R., Conrad M. (2021). Ferroptosis: Mechanisms, biology and role in disease. Nat. Rev. Mol. Cell Biol..

[13] Elumalai S., Karunakaran U., Moon J.S., Won K.C. (2022). Ferroptosis Signaling in Pancreatic β-Cells: Novel Insights & Therapeutic Targeting. Int. J. Mol. Sci..

[14] Sun L., Zong G., Pan A., Ye X., Li H., Yu Z., Zhao Y., Zou S., Yu D., Jin Q. (2013). Elevated plasma ferritin is associated with increased incidence of type 2 diabetes in middle-aged and elderly Chinese adults. J. Nutr..

[15] Wang X., Fang X., Zheng W., Zhou J., Song Z., Xu M., Min J., Wang F. (2021). Genetic Support of A Causal Relationship Between Iron Status and Type 2 Diabetes: A Mendelian Randomization Study. J. Clin. Endocrinol. Metab..

[16] Stancic A., Saksida T., Markelic M., Vucetic M., Grigorov I., Martinovic V., Gajic D., Ivanovic A., Velickovic K., Savic N. (2022). Ferroptosis as a Novel Determinant of β-Cell Death in Diabetic Conditions. Oxidative Med. Cell. Longev..

[17] Deng L., Mo M.Q., Zhong J., Li Z., Li G., Liang Y. (2023). Iron overload induces islet β cell ferroptosis by activating ASK1/P-P38/CHOP signaling pathway. PeerJ.

[18] Camunas-Soler J., Dai X.Q., Hang Y., Bautista A., Lyon J., Suzuki K., Kim S.K., Quake S.R., MacDonald P.E. (2020). Patch-Seq Links Single-Cell Transcriptomes to Human Islet Dysfunction in Diabetes. Cell Metab..

[19] Avrahami D., Wang Y.J., Schug J., Feleke E., Gao L., Liu C., Naji A., Glaser B., Kaestner K.H. (2020). Single-cell transcriptomics of human islet ontogeny defines the molecular basis of β-cell dedifferentiation in T2D. Mol. Metab..

[20] Xin Y., Kim J., Okamoto H., Ni M., Wei Y., Adler C., Murphy A.J., Yancopoulos G.D., Lin C., Gromada J. (2016). RNA Sequencing of Single Human Islet Cells Reveals Type 2 Diabetes Genes. Cell Metab..

[21] Lawlor N., George J., Bolisetty M., Kursawe R., Sun L., Sivakamasundari V., Kycia I., Robson P., Stitzel M.L. (2017). Single-cell transcriptomes identify human islet cell signatures and reveal cell-type-specific expression changes in type 2 diabetes. Genome Res..

[22] Son J., Ding H., Farb T.B., Efanov A.M., Sun J., Gore J.L., Syed S.K., Lei Z., Wang Q., Accili D. (2021). BACH2 inhibition reverses β cell failure in type 2 diabetes models. J. Clin. Investig..

[23] Segerstolpe Å., Palasantza A., Eliasson P., Andersson E.M., Andréasson A.C., Sun X., Picelli S., Sabirsh A., Clausen M., Bjursell M.K. (2016). Single-Cell Transcriptome Profiling of Human Pancreatic Islets in Health and Type 2 Diabetes. Cell Metab..

[24] Zhou N., Bao J. (2020). FerrDb: A manually curated resource for regulators and markers of ferroptosis and ferroptosis-disease associations. Database.

[25] Liberzon A., Subramanian A., Pinchback R., Thorvaldsdóttir H., Tamayo P., Mesirov J.P. (2011). Molecular signatures database (MSigDB) 3.0. Bioinformatics.

[26] Hänzelmann S., Castelo R., Guinney J. (2013). GSVA: Gene set variation analysis for microarray and RNA-seq data. BMC Bioinform..

[27] Cao J., Spielmann M., Qiu X., Huang X., Ibrahim D.M., Hill A.J., Zhang F., Mundlos S., Christiansen L., Steemers F.J. (2019). The single-cell transcriptional landscape of mammalian organogenesis. Nature.

[28] Garcia-Alonso L., Holland C.H., Ibrahim M.M., Turei D., Saez-Rodriguez J. (2019). Benchmark and integration of resources for the estimation of human transcription factor activities. Genome Res..

[29] Han H., Cho J.W., Lee S., Yun A., Kim H., Bae D., Yang S., Kim C.Y., Lee M., Kim E. (2018). TRRUST v2: An expanded reference database of human and mouse transcriptional regulatory interactions. Nucleic Acids Res..

[30] McEligot A.J., Poynor V., Sharma R., Panangadan A. (2020). Logistic LASSO Regression for Dietary Intakes and Breast Cancer. Nutrients.

[31] Engebretsen S., Bohlin J. (2019). Statistical predictions with glmnet. Clin. Epigenetics.

[32] Chen X., Ishwaran H. (2012). Random forests for genomic data analysis. Genomics.

[33] Miranda M.A., Macias-Velasco J.F., Lawson H.A. (2021). Pancreatic β-cell heterogeneity in health and diabetes: Classes, sources, and subtypes. Am. J. Physiol. Endocrinol. Metab..

[34] Saint-André V. (2021). Computational biology approaches for mapping transcriptional regulatory networks. Comput. Struct. Biotechnol. J..

[35] Tay Y., Rinn J., Pandolfi P.P. (2014). The multilayered complexity of ceRNA crosstalk and competition. Nature.

[36] Wajchenberg B.L. (2007). β-cell failure in diabetes and preservation by clinical treatment. Endocr. Rev..

[37] Xu E.E., Sasaki S., Speckmann T., Nian C., Lynn F.C. (2017). SOX4 Allows Facultative β-Cell Proliferation Through Repression of Cdkn1a. Diabetes.

[38] Miyatsuka T., Kosaka Y., Kim H., German M.S. (2011). Neurogenin3 inhibits proliferation in endocrine progenitors by inducing Cdkn1a. Proc. Natl. Acad. Sci. USA.

[39] Koyanagi A., Kotani H., Iida Y., Tanino R., Kartika I.D., Kishimoto K., Harada M. (2022). Protective roles of cytoplasmic p21(Cip1) (/Waf1) in senolysis and ferroptosis of lung cancer cells. Cell Prolif..

[40] Kang R., Kroemer G., Tang D. (2019). The tumor suppressor protein p53 and the ferroptosis network. Free. Radic. Biol. Med..

[41] Ding H., Wang F., Shi X., Ma H., Du Y., Hou L., Xing N. (2020). LncRNA MALAT1 induces the dysfunction of β cells via reducing the histone acetylation of the PDX-1 promoter in type 1 diabetes. Exp. Mol. Pathol..

[42] Liang Z., Wu Q., Wang H., Tan J., Wang H., Gou Y., Cao Y., Li Z., Zhang Z. (2022). Silencing of lncRNA MALAT1 facilitates erastin-induced ferroptosis in endometriosis through miR-145-5p/MUC1 signaling. Cell Death Discov..

[43] You L., Wang N., Yin D., Wang L., Jin F., Zhu Y., Yuan Q., De W. (2016). Downregulation of Long Noncoding RNA Meg3 Affects Insulin Synthesis and Secretion in Mouse Pancreatic Beta Cells. J. Cell. Physiol..

[44] Chen C., Huang Y., Xia P., Zhang F., Li L., Wang E., Guo Q., Ye Z. (2021). Long noncoding RNA Meg3 mediates ferroptosis induced by oxygen and glucose deprivation combined with hyperglycemia in rat brain microvascular endothelial cells, through modulating the p53/GPX4 axis. Eur. J. Histochem..

[45] Anandhan A., Dodson M., Shakya A., Chen J., Liu P., Wei Y., Tan H., Wang Q., Jiang Z., Yang K. (2023). NRF2 controls iron homeostasis and ferroptosis through HERC2 and VAMP8. Sci. Adv..

[46] Dai E., Meng L., Kang R., Wang X., Tang D. (2020). ESCRT-III-dependent membrane repair blocks ferroptosis. Biochem. Biophys. Res. Commun..

[47] Lin Z., Li W., Wang Y., Lang X., Sun W., Zhu X., Bian R., Ma Y., Wei X., Zhang J. (2023). SMSCs-derived sEV overexpressing miR-433-3p inhibits angiogenesis induced by sEV released from synoviocytes under triggering of ferroptosis. Int. Immunopharmacol..

[48] Yi J., Zhu J., Wu J., Thompson C.B., Jiang X. (2020). Oncogenic activation of PI3K-AKT-mTOR signaling suppresses ferroptosis via SREBP-mediated lipogenesis. Proc. Natl. Acad. Sci. USA.

[49] Fang X., Zhang J., Li Y., Song Y., Yu Y., Cai Z., Lian F., Yang J., Min J., Wang F. (2023). Malic Enzyme 1 as a Novel Anti-Ferroptotic Regulator in Hepatic Ischemia/Reperfusion Injury. Adv. Sci..

[50] Zhang W., Gong M., Zhang W., Mo J., Zhang S., Zhu Z., Wang X., Zhang B., Qian W., Wu Z. (2022). Thiostrepton induces ferroptosis in pancreatic cancer cells through STAT3/GPX4 signalling. Cell Death Dis..

[51] Abebe T., Mahadevan J., Bogachus L., Hahn S., Black M., Oseid E., Urano F., Cirulli V., Robertson R.P. (2017). Nrf2/antioxidant pathway mediates β cell self-repair after damage by high-fat diet-induced oxidative stress. JCI Insight.

[52] De Groef S., Renmans D., Cai Y., Leuckx G., Roels S., Staels W., Gradwohl G., Baeyens L., Heremans Y., Martens G.A. (2016). STAT3 modulates β-cell cycling in injured mouse pancreas and protects against DNA damage. Cell Death Dis..

[53] Weng Q., Zhao M., Zheng J., Yang L., Xu Z., Zhang Z., Wang J., Wang J., Yang B., Richard Lu Q. (2020). STAT3 dictates β-cell apoptosis by modulating PTEN in streptozocin-induced hyperglycemia. Cell Death Differ..

[54] Elghazi L., Bernal-Mizrachi E. (2009). Akt and PTEN: Beta-cell mass and pancreas plasticity. Trends Endocrinol. Metab..

[55] Zeng N., Yang K.T., Bayan J.A., He L., Aggarwal R., Stiles J.W., Hou X., Medina V., Abad D., Palian B.M. (2013). PTEN controls β-cell regeneration in aged mice by regulating cell cycle inhibitor p16ink4a. Aging Cell.

