# Supplementary material for: Single-Cell RNA-seq Analysis Reveals a Positive Correlation between Ferroptosis and Beta-Cell Dedifferentiation in Type 2 Diabetes
Source: Biomedicines. 2024 Jul 29;12(8):1687. doi: 10.3390/biomedicines12081687 (PMC11351120; doi:10.3390/biomedicines12081687)
Supplement: Supplementary file 1 [file biomedicines-12-01687-s001.zip › biomedicines-3113829-supplementary.pdf]

Supplementary Material

1 Supplementary Tables

Supplementary Table S1 Data sources

	ND donors	T2D donors	Cell Number	Platform
E-MTAB-5061	6	4	3355	SmartSeq2
GSE124742	18	7	6609	SmartSeq2
GSE154126	4	10	813	Fluidigm C1(SMART-seq)
GSE81608	12	6	1600	Fluidigm C1(SMART-seq)
GSE86469	5	3	638	Fluidigm C1(SMART-seq)
GSE98887	4	6	7155	Fluidigm C1(SMART-seq)

Abbreviations: ND, non-diabetic; T2D, type 2 diabetes mellitus.

2 Supplementary Figures

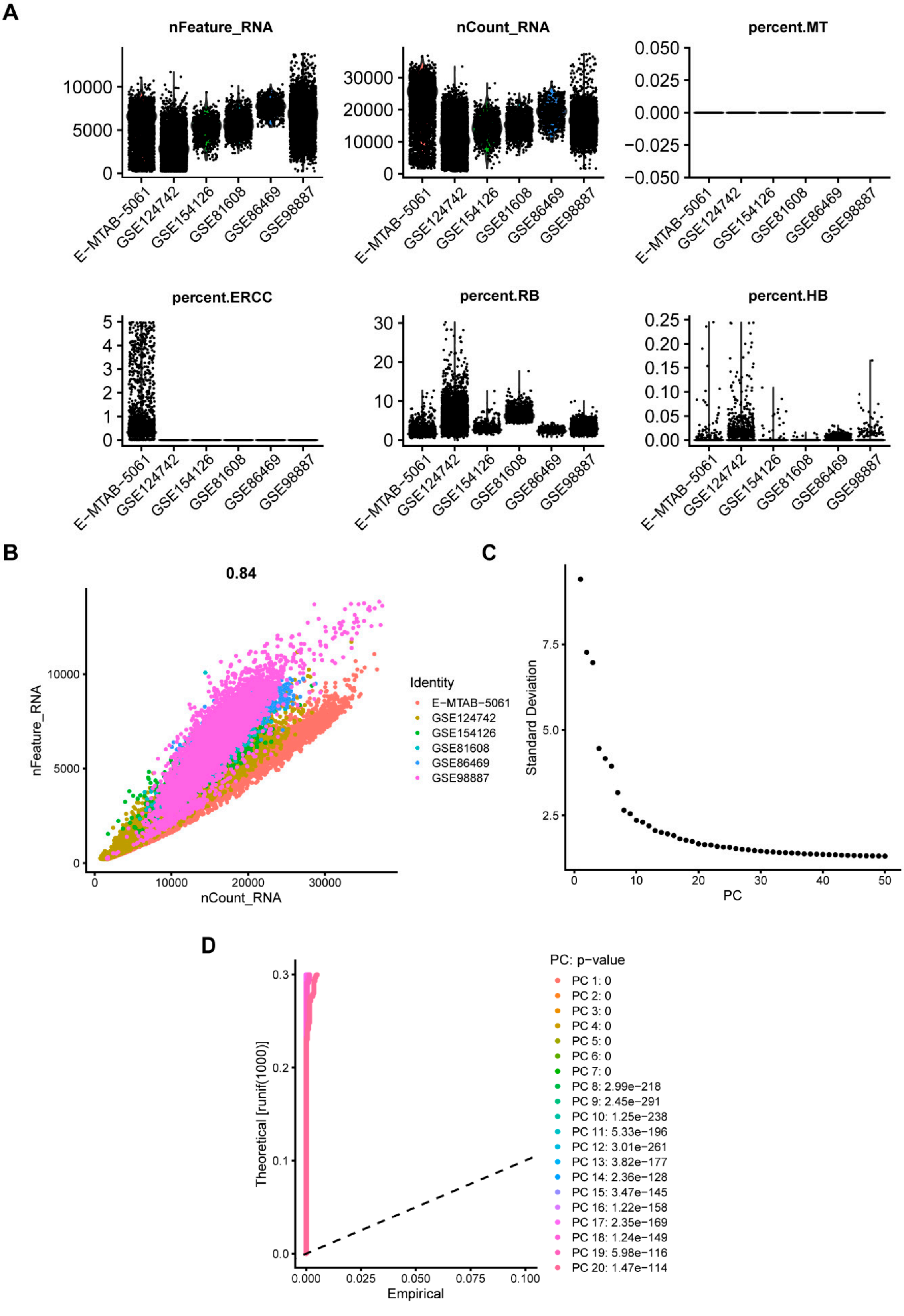
A
nFeature_RNA
nCount_RNA
percent.MT
percent.ERCC
percent.RB
percent.HB
E-MTAB-5061
GSE124742
GSE154126
GSE81608
GSE86469
GSE98887
B
0.84
nFeature_RNA
nCount_RNA
Identity
E-MTAB-5061
GSE124742
GSE154126
GSE81608
GSE86469
GSE98887
C
Standard Deviation
PC
D
Theoretical [runif(1000)]
Empirical
PC: p-value
PC 1: 0
PC 2: 0
PC 3: 0
PC 4: 0
PC 5: 0
PC 6: 0
PC 7: 0
PC 8: 2.99e-218
PC 9: 2.45e-291
PC 10: 1.25e-238
PC 11: 5.33e-196
PC 12: 3.01e-261
PC 13: 3.82e-177
PC 14: 2.36e-128
PC 15: 3.47e-145
PC 16: 1.22e-158
PC 17: 2.35e-169
PC 18: 1.24e-149
PC 19: 5.98e-116
PC 20: 1.47e-114

Supplementary Figure S1. Basic analysis procedure of the scRNA-seq data. **(A)** Quality control of the scRNA-seq data. (**B**) Relationship between gene numbers detected and expression values of genes in each dataset. (**C**) 20 significant principal components (PCs) identified based on the elbow plot of principal component analysis (PCA). (**D**) 20 PCs identified based on the JackStraw plot of PCA.

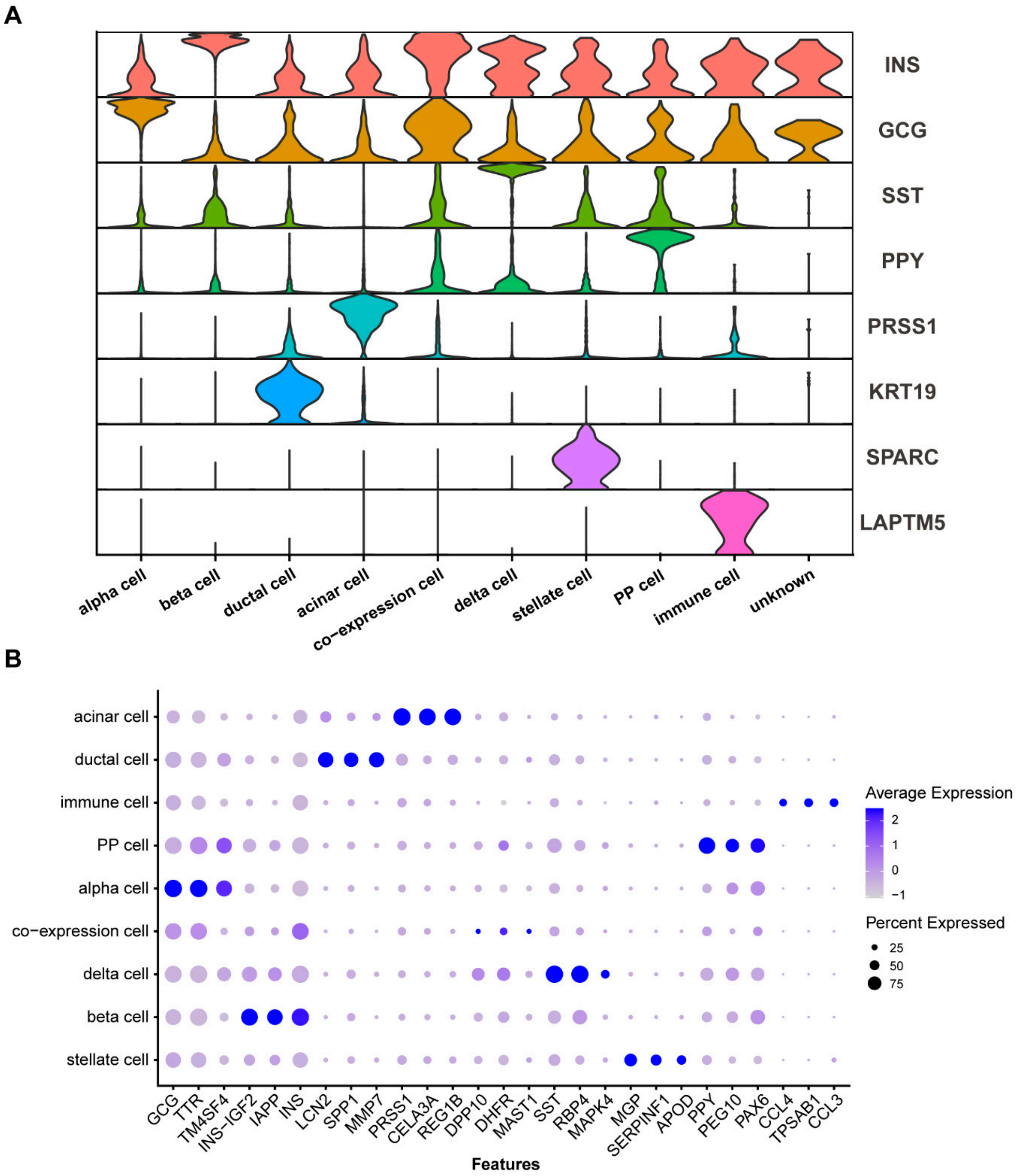

Supplementary Figure S2. Expression of markers in different types of endocrine and non-endocrine cells. **(A)** Violin plots showing specific markers in various cell types. **(B)** Dot map showing the top 3 markers in various cell types.

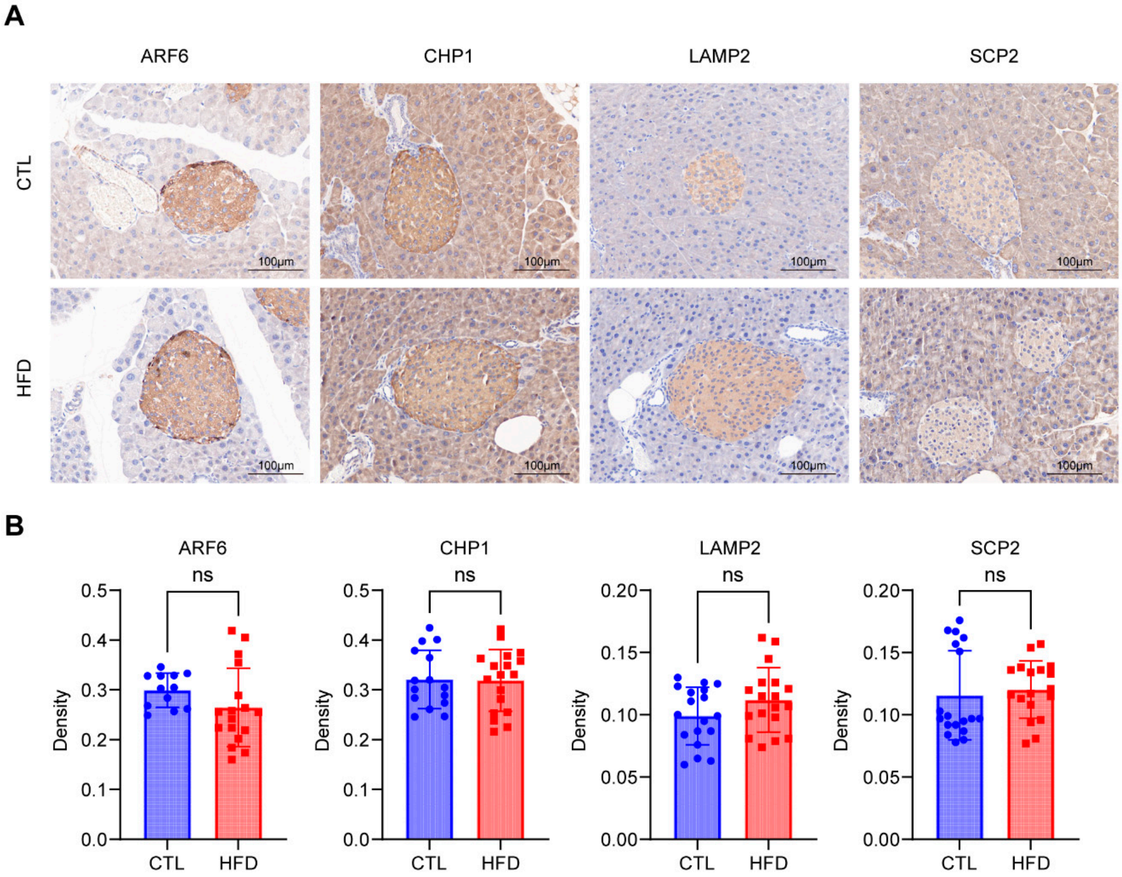

Supplementary Figure S3. Expression of genes with no significant changes between control and T2D mouse samples. **(A)** Immunohistochemical staining of ARF6, CHP1, SCP2, and LAMP2 protein in control and T2D mouse samples. **(B)** Quantitative analysis of immunohistochemical staining of ARF6, CHP1, SCP2, and LAMP2 protein (n = 3 in each group; 4-6 islets per mouse were analyzed; Values were shown as means ± standard deviation. *$P < 0.05$, **$P < 0.01$, ***$P < 0.001$ and ****$P < 0.0001$).
